# COVID-19 Preparedness Among Emergency Departments: A Cross-Sectional Study in France

**DOI:** 10.1017/dmp.2020.331

**Published:** 2020-09-10

**Authors:** Enrique Casalino, Donia Bouzid, Arsalene Ben Hammouda, Mathias Wargon, Sonja Curac, Romain Hellmann, Christophe Choquet, Daniel Aiham Ghazali

**Affiliations:** Assistance Publique-Hôpitaux de Paris (AP-HP), Hôpital Bichat, Emergency Department, Paris, France; Study Group for Efficiency and Quality of Emergency Departments and Non-Scheduled Activities Departments, Paris, France; Université de Paris, IAME, INSERM, F-75018 Paris, France; Hôpital de la Côte Fleurie, Normandie, France; Hôpital Delafontaine, Emergency Department, Saint Denis, France; Hôpital Beaujon, Emergency Department, Clichy, France; Regional Health Agency, Ile de France; Hôpital of Bichat and Beaujon, Emergency Medical Services, Paris, France

**Keywords:** COVID-19, emergency, preparedness, public health, responsiveness

## Abstract

**Objectives:**

The aim of this study was to evaluate hospital and emergency department (ED) preparedness in France facing the coronavirus disease 2019 (COVID-19) rapid growth epidemic-phase, and to determine the link between preparedness and responsiveness.

**Methods:**

In this cross-sectional study, from March 7 to March 11, 2020, all heads of ED departments in France were contacted to answer an electronic survey, including 23 questions. Quality, Organization, Training, Resources, Management, Interoperability, and Responsiveness were evaluated by calculating scores (10 points). Multivariate analysis of variance was used to compare scores. Spearman’s correlation coefficient and multifaceted regression analysis were performed between Responsiveness and dimensions scores.

**Results:**

A total of 287 of 636 French EDs were included (45.1%). Calculated scores showed (median): Quality 5.38; Organization 6.4; Training 4.6; Resources 4.13; Management 2.38; Interoperability 4.0; Responsiveness 6.25; seasonal influenza score was 5. Significant differences between scores as a function of hospital and ED main characteristics were found. Furthermore, we found significant correlations (*P* < 0.01) between Responsiveness and all preparedness dimensions. Organization (adjusted-R^2^ 0.2897), Management (aR^2^ 0.321), and Interoperability (aR^2^ 0.422) were significantly associated with Responsiveness.

**Conclusions:**

Preparedness in all its dimensions is low, indicating vulnerability. Preparedness and responsiveness face a certain and ongoing risk are close linked, and that Organizational, Management, and Interoperability dimensions are main determinants.

Biological outbreaks including pandemic influenza, new emerging infectious diseases^1,2^ and the situations of emergencies related to changing climate,^3^ represent a threat to public health. Among new infectious diseases risks, emerging viruse outbreaks including Ebola virus, severe acute respiratory syndrome (SARS), Middle East respiratory syndrome (MERS) diseases, and influenza pandemics have the potential to impose substantial mortality, morbidity, and economic burdens on human populations.^4,5^ Most of them are zoonotic viruses born from reservoir species.^5,6^ Seasonal influenza epidemics are also a major threat to emergency departments (ED) and hospitals and require an increase of hospital and intensive care unit beds.^7,8^

Since December 2019, SARS novel coronavirus-2 (SARS-nCov-2), responsible for coronavirus disease 2019 (COVID-19), has been reported in China.^9^ On January 24, 2020, France reported its first case,^10^ and on January 30, 2020, the World Health Organization (WHO) declared COVID-19 as the sixth public health emergency of international concern.^11^ At the time of writing this article, France has 10 transmission clusters, more than 10,000 proven cases, and all regions have declared cases with a rapid increasing trend in the number of confirmed cases, new cases, and deaths.^12^

In all countries and different health systems, a person with symptoms suggesting new epidemic risk will present to the ED.^13^ In all these cases, emerging infectious diseases and seasonal influenza epidemics, it is imperative to prevent further spread of the disease.^14,15^ EDs should be able to guarantee early detection and surveillance,^16^ apply isolation measures, and organize a triage system that predicates the correct orientation of the case regarding the patient’s severity and/or need for isolation in the medical and surgical wards of the hospital. Preparedness of health systems,^4,17,18^ hospitals and units of care,^2,16,19-21^ and EDs,^14,22,23^ has been assessed for the risks of outbreaks of new emerging infectious diseases, biological, and climate risks. Preparedness should be considered as a major determinant for preparing and responding to new risks.^14,24-27^ Recent evaluation of preparedness and response against diseases with epidemic potential in the European Union and United States has concluded that systems are vulnerable, and their capacities are reduced.^21,28^

In France, EDs are distributed in 636 hospitals: 473 public hospitals, 36 private nonprofit hospitals, and 127 private for-profit hospitals. Of these 636 health establishments, 547 receive adults, 73 receive both adults and pediatrics, and 16 receive exclusively pediatrics.^29^ Among the EDs, 59% receive less than 30,000 patients per year.^29^ The objective of the present study is to measure for COVID-19 the preparedness and the present-day response measures implemented by hospitals and EDs in France during the period of rapid growth in the number of cases of COVID-19 in France.

## METHODS

A cross-sectional study was undertaken of all EDs in France. On March 7, 2020, an electronic survey was distributed to heads of ED departments through solicitation by means of email. Emails were compiled by using the following lists: Study Group for Efficiency and Quality of Emergency Departments and Nonscheduled Activities Departments, and academic and hospitals associations. They received reminders for filling out the survey the next day and then every other day asking not to answer the survey if they had already done so. Heads of ED departments were asked to share the survey with nurse supervisors and physicians with responsibility for disaster response. The survey consisted of 23 questions including close-ended multiple select choices, linear scale, and open-ended questions. The survey was open for 5 d from March 7 to March 11, 2020.

The survey (Supplemental Appendix 1) was built to gather the required data. Its structure is based on the literature. Assessment of preparedness and responsiveness were derived from French,^30^ Centers for Disease Control and Prevention (CDC),^31^ and WHO^32^ recommendations. Questions were designed to cover some dimensions including management, resources, ED and hospital links, perceived risk and opportunities; planning, risk management, and infection control, and the main difficulties experienced by EDs; and to evaluate current tools and measures to respond to COVID-19 epidemic. These findings were validated by a committee of experts using the Delphi method, wherein questions were added, removed, or modified until a consensus of at least 65% agreement was reached.^33^ The experts are heads of ED departments who teach in a university degree in management of nonscheduled departments at the University of Paris. After validation, the survey was sent to the participants. We calculate 7 scores (i. Quality; ii. Organization; iii. Training; iv. Resources; v. Management; vi. Interoperability; vii. Responsiveness) by adding responses to selected questions. Score were normalized to 10 points.

### Data Analysis

Continuous variables are presented as mean ± SD and if necessary, as median and interquartile range (IQR), and categorical variables as number and percentage. We compared ED according to hospital type (academic, public, and private), ED type (adults and pediatrics), and ED activity (number of visits/year) (<30,000, 30,000, to 60,000, and >60,000). Comparison between the basic characteristics of respondent and nonrespondent EDs using a chi-squared test was carried out. To compare study groups, we used nonparametric Kruskal-Wallis for continuous variables and McNemar chi-squared test for categorical data. Multivariate analysis of variance (MANOVA) was used to compare scores as a function of ED and hospital characteristics. To evaluate the relationship between variables, we used first Spearman’s correlation coefficient (r) to determine the correlations among the variables. To evaluate the association between Responsiveness and Preparedness dimensions, a multifaceted regression analysis was performed between Responsiveness score and hospital and ED characteristics, and Quality, Organization, Training, Resources, Management and Interoperability scores, as well as hospital and ED characteristics. We selected the relevant variables based on the adjusted R^2^, with a stepwise approach.

*P* values were 2-tailed, and for each analysis, *P* value of <0.05 was considered significant. All statistical analysis was conducted using Statistica v12 software.

All data was completely anonymous and currently used for quality evaluation. Study was conducted in accordance with the 1964 Helsinki Declaration. The researchers received ethical approval to conduct this study by the institutional board.

## RESULTS

We collected 310 responses. We eliminated duplicates and kept primarily the response of the manager, the nurse supervisor, and if necessary, of a team physician. Finally, 287 EDs of the 636 French hospital responded (45.1%). Among them, 71 were academic (24.7%), 149 public (51.9%), and 67 private (23.3%); 245 EDs receive exclusively adults (85.7%) and 42 pediatrics and adults or pediatrics (14.3%); 74 (25.8%) receive less than 30,000, 140 (48.8%) between 30,000 and 60,000, and 73 EDs (25.4%) more than 60,000 visits a year. Nonrespondent EDs were distributed in 253 (72.5%) public hospitals and 96 (27.5%) private hospitals. There was no difference in the distribution between respondent and nonrespondent EDs (*P* = 0.23). Of the 349 nonrespondent EDs, 302 (86.5%) receive adults, 47 (13.5%) receive both adults and pediatrics, or exclusively pediatrics. There was no difference between respondent and nonrespondent EDs (*P* = 0.67). On the other hand, 301 (86.2%) nonrespondent hospitals receive less than 30,000 and 48 (13.8%) more than 30,000 visits a year, respectively. Respondent EDs have significantly higher annual activity than nonresponding emergency departments (*P* < 0.001).

### ED Preparedness

Table 1 presents the responses to ED Preparedness evaluation as a function of hospital type. We note that the EDs consider that their mission is the identification, reception, and management of suspected cases of COVID-19 with signs of severity (7.1 ± 3.5), but they are more reluctant to receive the cases without signs of severity (2.9 ± 2.7). We see differences between types of hospitals: the academic are significantly in favor of welcoming patients with signs of severity, and the private more in favor of welcoming patients without signs of severity. We found that only 58/87 (20.2%) of EDs feel able to ensure these missions, and 89/287 (31%) to support these patients despite the crowding in the ED, with differences between types of hospitals revealing the private and public feel mostly the inability to conduct these missions. Furthermore, we found that 86/287 (30%) and 92/287 (32.1%) think they can ensure the safety of COVID-19 patients and other patients, respectively, with significant differences between groups. While 278/287 (96.7%) report that they train their teams for COVID-19 management, the training of doctors and nurses is deemed insufficient by 29/287 (10.1%) and 17/287 (5.9%), mainly among private (20.9%). This explains a low security score for health-care workers (HCW) (6.0 ± 2.8), especially in private, and the feeling of endangering team members at 179/287 (62.4%), mainly among private.

**TABLE 1:** ED Preparedness as a Function of Hospital Characteristics

	Type of Hospital Structure			*P*-Value
	Academic	Public	Private	
	*n* = 71	*n* = 149	*n* = 67	
COVID-19 without severity criteria it is our mission, mean (SD)	2.15 (2.58)	2.74 (2.26)	3.88 (2.58)	0.001
COVID-19 with severity criteria It is our mission, mean (SD)	9.56 (1.53)	5.89 (3.75)	7.12 (3.17)	0.00 0 1
We will be able to carry out these missions without any problems, N° (%)	43 (60.6)	15 (10.1)	0 (0)	0.00001
We are overwhelmed, it’s difficult if not impossible, N° (%)	1 (1.4)	49 (32.9)	39 (58.2)	0.000001
COVID-19 cases security is guaranteed in our ED, mean (SD)	7.17 (2.52)	6.04 (2.54)	2.52 (1.8)	0.00001
You think it will have a decrease in quality of care for COVID-19 patients (yes), N° (%)	29 (40.9)	44 (29.5)	13 (19.4)	0.02
You think it will have a decrease in quality of care for other patients (yes), N° (%)	15 (21.1)	63 (42.3)	14 (20.9)	0.0006
Usual ED crowding is a main problem, N° (%)	18 (25.4)	117 (78.5)	41 (61.2)	0.000001
Too many difficulties in hospital bed management, N° (%)	43 (60.6)	131 (87.9)	66 (98.5)	0.000001
HCW received a training program for COVID-19 and to PPE, N° (%)	69 (97.2)	143 (96)	66 (98.5)	0.6
Insufficient training of doctors, N° (%)	0 (0)	15 (10.1)	14 (20.9)	0.0003
Insufficient training of nurses, N° (%)	2 (2.8)	1 (0.7)	14 (20.9)	0.000001
HCW security is guaranteed in our ED, mean (SD)	7 (2.16)	6.36 (2.42)	4.16 (2.82)	0.0001
Will endanger the safety of ED HCW, N° (%)	14 (19.7)	99 (66.4)	66 (98.5)	0.00001
May be responsible for the departure of some HCW, N° (%)	13 (18.3)	30 (20.1)	26 (38.8)	0.005
Our premises are adapted to COVID-19 care, mean (SD), N° (%)	4.28 (3.02)	5.56 (3.77)	4.96 (2.6)	0.4
No problem with premises, N° (%)	40 (56.3)	56 (37.6)	13 (19.4)	0.00005
No ED room to isolate a suspicious case with signs of severity, N° (%)	2 (2.82)	18 (12.1)	15 (22.4)	0.002
No ambulatory area to isolate suspected cases without signs of severity, N° (%)	16 (22.5)	16 (10.7)	27 (40.3)	0.000001
Narrow premises and/or insufficient surface area, N° (%)	29 (40.9)	32 (21.5)	28 (41.8)	0.001
Not enough ED rooms, N° (%)	30 (42.3)	46 (30.9)	26 (38.8)	0.2
Possibility to differentiate “isolation (or dirty)” and “clean” circuits, N° (%)	29 (40.9)	31 (20.8)	0 (0)	0.000001
Our nurses/nurses assistants team number is enough, mean (SD)	6.01 (2.46)	5.87 (1.23)	7.81 (1.33)	0.00001
We have no nurse staff difficulties, N° (%)	1 (1.4)	16 (10.7)	0 (0)	0.001
Nurses must work overtime, N° (%)	44 (62)	103 (69.1)	54 (80.6)	0.05
Nurses will be assigned to ED from non-priority ward/ambulatory areas, N° (%)	28 (39.4)	58 (38.9)	27 (40.3)	0.98
Our physicians team number is enough, mean (SD)	4.51 (3.66)	7.04 (1.47)	7.3 (1.38)	0.00001
We have no physician staff difficulties, N° (%)	13 (18.3)	2 (1.34	0 (0)	0.000001
Physicians must work overtime, N° (%)	70 (98.6)	105 (70.5)	53 (79.1)	0.00001
Physicians will be assigned to ED from non-priority wards/ambulatory areas, N° (%)	15 (21.1)	43 (28.ç)	14 (20.9)	0.3
Insufficient team management, N° (%)	27 (38)	15 (10.1)	13 (19.4)	0.00001
Resistance to organizational change, N° (%)	14 (19.7)	43 (28.86)	13 (19.4)	0.2
Lack of time to drive organizational change and leadership, N° (%)	16 (22.5)	86 (57.72)	27 (40.3)	0.000001
Lack of cooperation with GP and ambulatory care, N° (%)	70 (98.6)	103 (69.1)	27 (40.3)	0.000001
Usual bed management is a main problem, N° (%)	71 (100)	149 (100)	67 (100)	NC
Lack of cooperation with medical surgical ward, N° (%)	71 (100)	149 (100)	67 (100)	NC

ED premises are not considered suitable for the reception of COVID-19 patients (5.1 ± 3.2), and this in all kinds of establishments. Only 109/287 (38%) report that their premises are suitable to handle COVID-19 cases, but again with significant differences between groups. We found that 35/287 (12.2%) are not able to put into isolation COVID-19 patients with severity signs, but the figure is 22.9% of private, and that 59/287 (20.6%) cannot set up an ambulatory sector for patients without signs of severity, and this figure reaches 40.3% in the private. We note that 89/287 (31%) and 102/287 (35.5%) say their area and number of rooms are inadequate. A total of 60/287 (20.9%) say that their structures do not allow them to create distinct circuits for COVID-19 cases. EDs declare that the number of nurses (6.4 ± 1.8) and physicians (6.5 ± 2.5) are adequate, with significant differences between groups. EDs note that the main response to the lack of staff is the increase in the working time of their staff of nurses 201/287 (70%) and physicians 228/287 (79.4%). EDs also indicate that they require a reallocation of physicians and nurses from other nonpriority areas of the hospital in 113/287 (39.4%) and 228/287 (79.4%), respectively. Some management deficiencies were pointed out, including insufficient team management, resistance to organizational change, and lack of time to drive organizational change and leadership, with different frequencies (10% to 40%). Lack of cooperation with general practitioners (GPs) and ambulatory care is less frequent in private (40%) than in academic (99%) and public (69%), and 100% indicates that usual bed management and lack of cooperation with medical/surgical wards of the hospital and trust are the main problems.

### Evaluation of EDs During Seasonal Influenza Epidemics

We found that EDs considered that quality of care is not provided by the hospital and the ED during the seasonal influenza epidemic period (4.65 ± 2.8) (median 5 [IQR 2-7]). They considered that the main explanations are the unsuitable structures of ED (119/287 [41.5%]), an insufficient number of medical and nurses staff (169/287 [58.9%]), the need to improve the organization of ED (182/287 [63.4%]), the hospital’s inability to manage the circuits of scheduled and nonscheduled hospitalizations (258/287 [89.9%]), and the need to review the current isolation procedures and to increase staff awareness of personal protection procedures (207/287 [72.1%]). By using correlation models between seasonal influenza preparedness score and COVID-19 Preparedness dimensions we found: Quality (r = −0.17; *P* = 0.006), Organization (r = −0.68; *P* = 0.01), Training (r = −0.45; *P* = 0.0001), Resources (r = 0.27; *P* = 0.7), Management (r = −0.35; *P* = 0.0001), Interoperability (r = −0.8; *P* = 0.2), and Responsiveness (r = −0.47; *P* = 0.0001).

### ED and Hospital Response Measurements

Table 2 presents the responses put in place by EDs and hospitals facing COVID-19. We find that 81% (private) to 100% (academic) of EDs have implemented the early identification and placement of surgical masks from the reception of ED for all patients with respiratory symptoms. The identification of rooms dedicated to suspected COVID-19 patients ranges from 79% (academic and private) to 87% (public), and the establishment of an identified circuit for suspected COVID-19 cases without a sign of severity from 80% (academic) to 100% (private). Isolation measures for all suspected cases in the ED and observation unit and through ED-admitted patients have been put up mainly by the academic and public (100% and 98.7%, 98.6% and 77.2%), while that in private is only 59.7% and 77.6%. The ED guidelines were drafted by 78.9% to 100% of ED, but their validation by ED management staff and presentation to the team was less common in academic than in public and private. The hospital has set up dedicated circuits for patients suspected of COVID-19 without (157/287 [54.7%]) and with (126/287 [43.9%]) severity criteria, in both cases less frequently in academic than in private and public. A total of 132/287 (46%) of hospitals plan to increase the number of medical and surgical wards (MSW) rooms dedicated to COVID-19 cases. Public engagement is less than that of academic and private.

**TABLE 2:** Responsiveness Facing the COVID-19 Pandemic as a Function of Hospital Characteristics

	Type of Hospital Structure			*P*-Value
	Academic	Public	Private	
	*n* = 71	*n* = 149	*n* = 67	
ED				
Surgical mask upon ED arrival for any patient with respiratory symptoms, N° (%)	71 (100)	148 (98.3)	54 (80.6)	0.000001
Identification of boxes dedicated to suspected cases of COVID-19, N° (%)	56 (78.9)	130 (87.3)	53 (79.1)	0.17
Dedicated circuit for cases without severity criteria, N° (%)	57 (80.3)	134 (89.9)	67 (100)	0.0006
Isolation measures for all suspected cases in ED and observation unit, N° (%)	71 (100)	147 (98.7)	40 (59.7)	0. 00001
Isolation measures for all through ED admitted patients, N° (%)	70 (98.6)	115 (77.2)	52 (77.61)	0.0002
ED guidelines for COVID-19 cases management, N° (%)	56 (78.9)	147 (98.67)	67 (100)	0.000001
ED COVID-19 guidelines validated by medical or quality director, N° (%)	53 (80.3)	119 (79.87)	66 (98.5)	0.001
ED guidelines presented to ED Team, N° (%)	41 (57.8)	117 (78.5)	66 (98.5)	0.000001
Presentation of guidelines to ED Team (in training meetings), N° (%)	41 (57.8)	73 (49)	53 (79.1)	0.0002
Hospital				
Circuit outside ED for COVID-19 cases without severity criteria, N° (%)	29 (40.9)	89 (59. 7 3)	39 (58.2)	0.03
Circuit outside ED for COVID-19 cases with severity criteria, N° (%)	16 (22.5)	58 (38.93)	52 (77.6)	0.000001
Increases number of rooms in medical surgical wards dedicated to COVID-19, N° (%)	41 (57.8)	50 (33.6)	41 (61.2)	0.00006

### Preparedness and Responsiveness Relationships

The composite scores calculated on the different dimensions assessed are presented in Table 3. By using MANOVA, we found that type of hospital (academic, public, private), type of ED (pediatrics and adults) and the size of ED (<30,000, 30,000 to 60,000, >60,000) were associated with differences in the different scores. Overall, adult sites, academic and ED with over 60,000 visits have better scores than others.

**TABLE 3:** Preparedness Scores Values Facing the COVID-19 Pandemic as a Function of Hospital and ED Characteristics

	All Study Questionnaires (*n* = 287)					MANOVA																		
										Pediatric		Adults		*P*-Value	Academic		Public		Private		*P*-Value	<30 000		30000 to 60000		>60 000		*P*-Value
	Mean	SD	Median	IQR		Mean	SD	Mean	SD	Mean	SD	Mean	SD		Mean	SD	Mean	SD	Mean	SD		Mean	SD					
Quality	4.48	1.3	5.38	3.8	5.55	5.53	0.02	4.31	1.3	0.03	5.15	0.7	4.28	1.4	4.22	1.2	0.00001	4.44	1.3	4.14	1.3	5.17	0.7	0.000001
Organization	6.57	1.8	6.4	5.23	8.74	6.41	1.9	6.59	1.7	0.4	6.96	2.1	6.66	1.8	5.95	1.2	0.3	5.28	1.1	6.58	1.7	7.85	1.6	0.0000001
Training	4.27	1	4.6	4.6	4.6	4.60	0.0	4.22	1.1	0.1	4.54	0.4	4.38	0.7	3.76	1.6	0.03	3.84	1.6	4.37	0.7	4.54	0.4	0.01
Resources	4.18	1.2	4.13	3.38	5	3.23	1.1	4.34	1.1	0.0000001	4.33	1.1	4.13	1.2	4.15	1.1	0.0002	4.04	1.2	4.28	1.3	4.14	0.8	0.6
Management	1.94	0.7	2.38	1.5	2.47	1.93	0.5	1.94	0.7	0.8	1.93	0.5	1.88	0.8	2.08	0.5	0.03	1.96	0.5	1.84	0.7	2.10	0.8	0.005
Interoperability	4.46	1.8	4	4	6	4.00	1.7	4.54	1.8	0.02	3.69	1.7	4.52	1.3	5.13	2.4	0.0000001	3.32	1.4	5.06	1.7	4.47	1.7	0.0000001
Responsiveness	5.81	2.5	6.25	3.75	8.75	4.94	2.7	5.96	2.5	0.8	5.77	2.6	5.68	2.6	6.16	2.4	0.0000001	3.34	2.1	6.63	2.0	6.75	2.3	0.0000001

**FIGURE 1:**
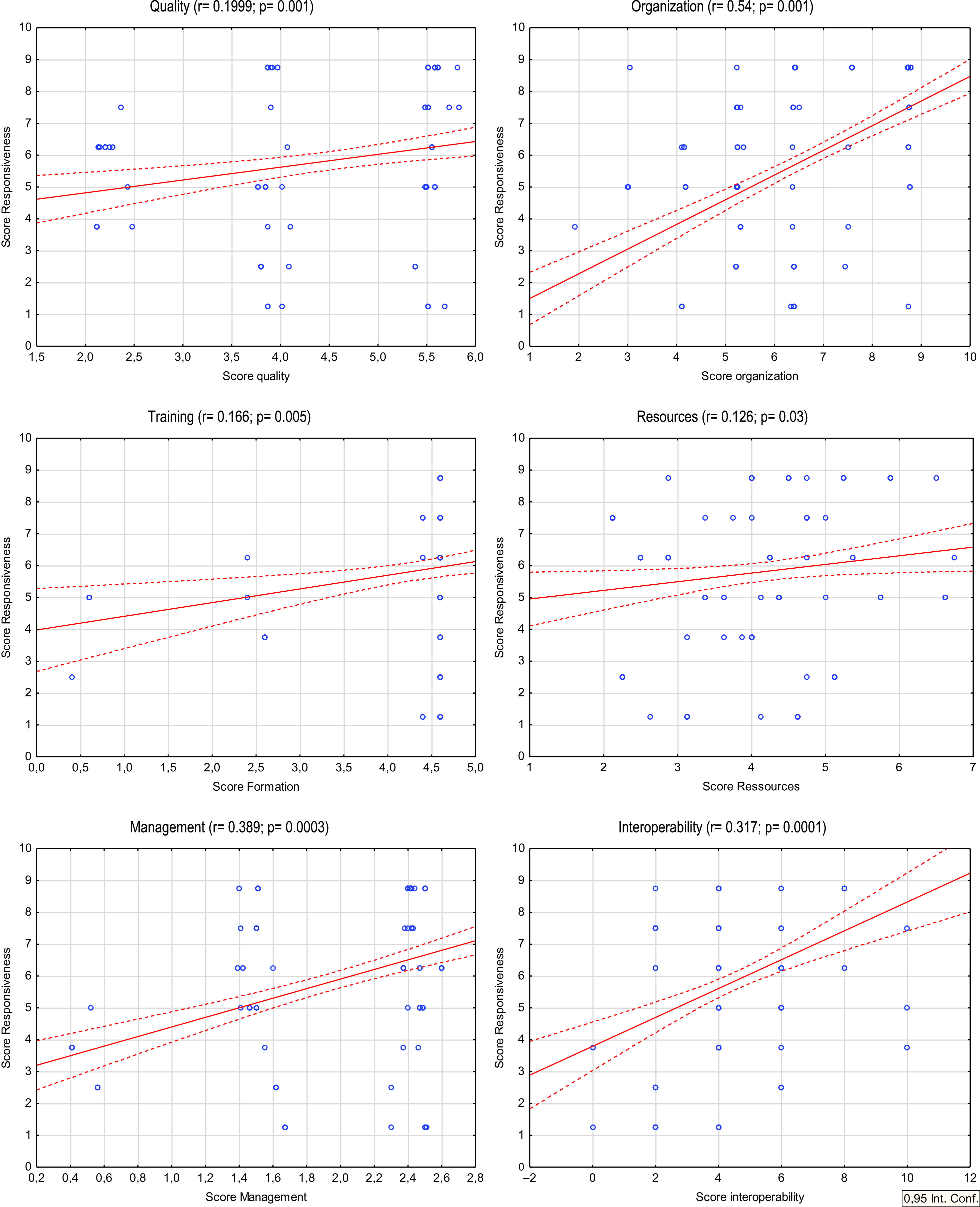
Relationship Between Responsiveness and Preparedness Scores Facing the COVID-19 Pandemic.

We found that the Responsiveness score was significantly associated with the different dimensions calculated scores (Figure 1). In multifaceted regression analysis, we found that Organization (adjusted R^2^ 0.2897; *P* = 0.000001), Management (R^2^ 0.321; *P* = 0.002), and Interoperability (R^2^ 0.422; *P* = 0.000001) were significantly associated with Responsiveness. Of interest, hospital and ED characteristics were not associated with Responsiveness.

## DISCUSSION

Nearly half of the health establishments responded to this survey, with a similar distribution and type of activity between public and private EDs within respondent and nonrespondent hospitals. However, it was mainly the EDs with the largest annual activities that responded. Our study indicates that hospital and ED preparedness and response capacity during the COVID-19 rapid growth epidemic phase, is low, pointing out a vulnerable system. Academic, public, and private hospitals and their EDs do not have the same perceived difficulties and the same response capacities. Furthermore, we found weak values for all the dimensions assessed (Quality, Organization, Training, Resources, Management, Interoperability, and Responsiveness) and that the characteristics of hospitals and EDs were associated with significant differences. Evermore, we found significant relationships between Responsiveness and Organization, Management, and Interoperability scores, indicating that these features should be considered to promote ED and hospital preparedness and response capacity performing programs.

We found that hospital preparedness was different as a function of hospital type (academic, public, and private). We found that their experiences, missions, organizations, perceived difficulties, and risks, but also their tools in place to tackle the challenge of COVID-19, were different depending on hospital type. Team training management for cases of COVID-19 was carried out by more than 98% of EDs, with no difference between types of hospital. However, the private more often consider this training insufficient. The public and private more often report difficulties on hospital and ED organization, bed management, unsuitable structures to receive COVID-19 patients, and the inadequate number of medical and nurse staff. These differences may explain why academic are more inclined to think that they can receive cases of COVID-19 and other patients without a loss in the quality of care and to think that their staff is safe. But also, to consider that welcoming COVID-19 cases with signs of severity is their mission. We also note that ED crowding and bed management insufficiencies are stronger in public and private, and that this probably weighs in the ability to integrate the possibility of an additional activity linked to an outbreak, such as is a mission of the ED.

The preparedness of hospitals and EDs has been assessed in the face of risks already realized, and on the theoretical capacity of ED to set up tools to respond to these situations based on guidelines.^16,17,26^ We evaluated here the preparedness and the current response capacity of the ED and hospital as they face a new viral outbreak at the time of the rapid epidemic growth of COVID-19. In addition, the COVID-19 epidemic occurred during the decreasing phase of the number of new cases of seasonal influenza,^34^ and while the ED and hospital teams are coming out of a period of overactivity during the winter season linked to the influenza epidemic.^7,8^ The perception of EDs could have been modified by this context. Our study shows that EDs consider that the quality of care is not ensured by the hospital and the ED during seasonal influenza epidemics. Hospital and ED preparedness during previous seasonal influenza epidemic periods was associated with ED preparedness for COVID-19. Otherwise, EDs indicated that the main explanations for this inability to ensure the quality and safety of patient care are the unsuitable structures of EDs, an insufficient number of medical staff and nurses, the need to improve the organization of EDs, and the inability of the hospital to manage the circuits of scheduled and unscheduled hospitalizations. They also emphasize the need to review the procedures for influenza isolation and strengthen staff awareness of patient and caregiver precaution measures. These results are close to those we obtained for COVID-19 and show that the difficulties identified are usual and that this compromises the ED response capacity to a new viral risk. It has been recently suggested that influenza preparedness provides some lessons for future pandemics.^35^

We found that the obtained values for the Preparedness dimensions are generally low. The Quality score shows the fears of EDs of not being able to ensure the quality of care and the safety of patients and HCW. The Organization score indicates the difficulties faced by the EDs, mainly the frequent ED overcrowding, the difficulties related to inadequate structures to face this new challenge. Although EDs have implemented specific COVID-19 training, the Training score reflects the oppositions regarding these trainings and fears concerning the safety of hospital and ED HCW. This is to be compared with the Resources score, which evaluated the ED staff. The EDs consider their staff inadequate in number, insufficiently trained in new risks, and see that the main response to overcome these shortcomings will be the increase in working hours with an increased risk of error, which weighs on the quality of care and on staff safety. The score Management reflects the perceived difficulties to identify ED missions and to create an ED Team. It indicates how difficult it can be to form management teams and the possibility of leading change projects. The Interoperability score indicates that EDs encounter difficulties in creating functional links with ambulatory care and general practitioners (GP), and with the hospitalization sectors of their own hospital and trust. Responsiveness score measures the ability of EDs to put in place the necessary tools to meet current organizational circuits to ensure the quality of patients care and protection.

The type of ED (pediatric vs adult), hospital type (academic, public, private), and the size of the ED, have been associated in multivariate analysis with significant differences for most of these scores, indicating that hospital and ED characteristics were associated with hospital and ED preparedness. Otherwise, we found significant relationship between Responsiveness and all preparedness dimensions (Quality, Organization, Training, Resources, Management, Interoperability). These results suggest that preparedness dimensions, notably those with greater r values (Organization, Management, and Interoperability), are close related to Responsiveness. Even more by using multifaceted analysis we showed that Organization, Management, and Interoperability were significantly associated with Responsiveness. Obtained adjusted R^2^ indicate that these dimensions are of great importance, because the link between preparedness and responsiveness has been demonstrated.

### Limitations

The present study is not without limitations. First, this survey is on a voluntary basis and not all EDs responded. Respondents may be the most involved in crisis management. On the other hand, the respondent EDs are those with the most activity. It also bears mentioning that the activity related to the COVID-19 pandemic is unknown to the nonrespondent EDs. However, nearly half of the EDs responded to the survey with no difference in distribution between respondents and nonrespondents in terms of adult or pediatric activity and distribution between public and private services. As a result, the impact of this bias is mitigated. It is possible that opinions and preparation were changing over the 5-d course of the survey due to the daily increase in COVID-19 activity. There is a lack of data on nonresponding EDs on this point. Finally, already affected EDs might have had a different point of view from those who were not.

## CONCLUSIONS

The strength of this work is to have assessed preparedness and responsiveness during the actual COVID-19 epidemic episode. French hospitals and EDs are concerned about the foreseeable ED COVID-19 related overactivity, and that preparedness in all its dimensions is low, indicating vulnerability. Our results demonstrate that preparedness and responsiveness are closely related, and that Organizational, Management, and Interoperability are main determinants. Our study suggests that preparedness should be evaluated to warrant hospital and ED responsiveness. But also, that proposal to improve preparedness and responsiveness might consider these dimensions and the characteristics of the concerned hospital and ED. To optimize the ED and hospital preparedness and responsiveness, we need to ameliorate the ED and hospital functioning during seasonal influenza epidemics. The strength of the present study is to assess preparedness and responsiveness to a current risk, not to a theoretical or previous risk. Nevertheless, our results cannot predict the adaptability and resilience of our hospitals and EDs.

## Data Availability

Data for this article are included in the online supplementary material.

## Supplementary Material

To view supplementary material for this article, please visit https://doi.org/10.1017/dmp.2020.331
